# Critical Levels of Ozone Over the United Kingdom: Mapping Aggregate Exceedances Over Moderate to High Thresholds

**DOI:** 10.6028/jres.099.033

**Published:** 1994

**Authors:** R. I. Smith, C. W. Anderson, D. Fowler

**Affiliations:** Institute Terrestrial Ecology, Edinburgh Research Station, Bush Estate, Penicuik, Midlothian EH26 0QB, Scotland; University of Sheffield, School of Mathematics and Statistics, P.O. Box 597, Sheffield, S10 2UN, England; Institute Terrestrial Ecology, Edinburgh Research Station, Bush Estate, Penicuik, Midlothian EH26 0QB, Scotland

**Keywords:** aggregate excess distribution, critical level, mapping, ozone

## Abstract

The critical level for ozone, above which it has a detectable effect on bio-logical targets, is potentially to be set by the United Nations Economic Commission for Europe at 300 nL·h/L hours per annum over 40 nL/L. It is therefore important to determine the aggregate exccedancc over 40 nL/L throughout the United Kingdom. Over most of the UK, ozone concentrations are unknown so we rely on our understanding of the atmospheric processes and on the statistical properties of ozone concentrations to interpolate between monitoring sites. This paper describes the application of statistical models derived for storm severity data to the ozone data for the United Kingdom. Aggregate excess distributions were fitted to data from all rural monitoring sites using a Weibull model with a 40 nL/L threshold. At this threshold the scale parameter has a spatial interpretation, but, with higher thresholds, there were problems with missing data and small scale spatial effects were not detected. The approach appears successful for all except very large aggregate exceedances which deviate from the Weibull predictions.

## 1. Introduction

The major public concern with ozone, O_3_, in Europe has focused recently on the existence of “ozone holes” in the stratosphere caused by the depletion of ozone as a consequence of chlorofluorocarbon emissions. Ozone is also present in the troposphere and in the planetary boundary layer at concentrations, i.e., volume fractions between 10 and 200 nL/L (i.e., parts per billion, ppb = 10^−9^). In the second half of the last century European mean concentrations ranged between 10 ppb and 15 ppb [8]. Current mean concentrations are about twice these values and ozone episodes with peak concentrations between 100 ppb and 200 ppb occur, a level known to cause damage to many plant species. Episodes happen if the precursor gases for photochemical ozone production (oxides of nitrogen, NO and NO_2_, and volatile organic compounds, VOCs) are present in suitable meteorological conditions for the chemical reactions to occur (ideally hot summer days with clear skies and low wind speeds).

**Fig. 1:**
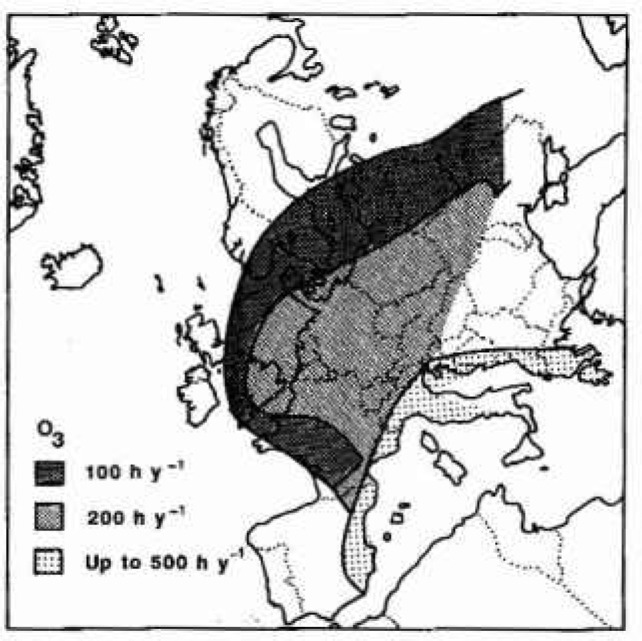
Hours when ozone exceeds 60 ppb.

The description of spatial patterns in exposure of vegetation to ozone over Europe has been hampered by the limited availability of monitoring data, the very large spatial variability in ozone concentrations and a poor understanding of the underlying mechanisms regulating the ozone exposure of terrestrial ecosystems. Defining a threshold for phytotoxicity is not simple. However, at 60 ppb of ozone there is little doubt that there is a clear contribution from photochemical production in polluted air and a map of hours over 60 ppb for Europe (Fig. 1) is a guide to some broad trends [4]. In a large area north of the Alps, covering most of Germany and parts of neighbouring countries, 200 hours per year above 60 ppb is common. North and west of this area the annual duration of exposure declines but to the east there is so little information available that mapping is uncertain. The Mediterranean zone of high ozone exposure reflects recent work showing that ozone episodes are common events in this region but the levels have not yet been well quantified.

Although the meteorological conditions leading to ozone episodes are similar in a general sense at all sites, the climates of northern and southern Europe lead to very different patterns of events. In northern Europe, typical episodes occur when a stationary spring or summer anticyclone provides the conditions for ozone production from the emitted precursor gases to add appreciably to the background concentration of about 30 ppb. Typical production rates give net increases of 10 ppb to 20 ppb per day and a succession of 8 to 10 such days leads to peak concentrations of 150 ppb to 200 ppb. Often in northern Scandinavia, Britain, Ireland and western France the ideal meteorology exists but in the absence of upwind precursors. In Germany and central Europe almost all wind directions provide the precursors and hence the NW-SE gradient in episodes. In southern Europe, the meteorological conditions are more stable and episodes can occur every day for long periods. However the effects of both sea breezes and the development of intense thermal low pressure areas on the air circulation causes very variable patterns of ozone exposure.

**Fig. 2:**
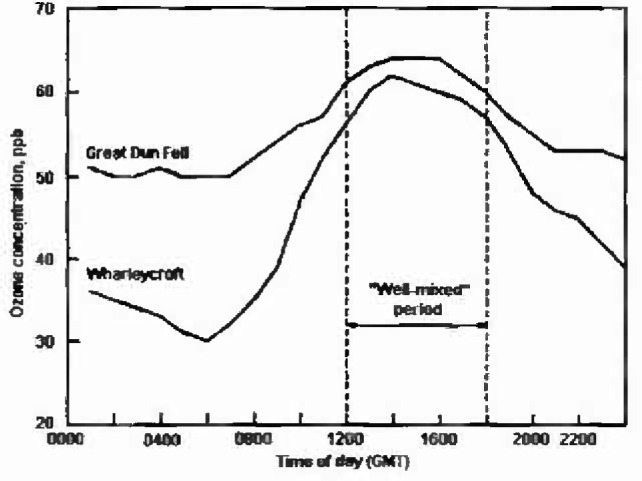
Typical altitude effect on the diurnal cycles of ozone concentration.

Superimposed on this two dimensional surface there is a daily cycle in ozone concentration which is a very important and variable feature. At low altitude inland sites a marked diurnal variation (of the order of 30 ppb) is observed but at high elevation the amplitude of the diurnal cycle gradually reduces to less than 5 ppb at mountain tops. Figure 2 shows data for 1 day at both Great Dun Fell (847 m above sea level) and Wharleycroft (206 m above sea level), two sites which are less than 10 km apart. Hill tops are generally windy sites at which the terrestrial surfaces are well connected to the free troposphere and where the downward supply of ozone to the surface exceeds the rate of deposition. At low level sites the thermal stratification of the atmosphere with the development of a nocturnal inversion restricts the supply of ozone from above during the night and morning. In these conditions both deposition to the surface and the nocturnal atmospheric chemical titration of ozone with nitric oxide causes the surface concentrations of ozone to decline, potentially to negligible levels. At coastal sites the effects of land and sea breezes strongly modify the ozone exposure of the ground.

The main concern with rural ground level ozone concentrations is the damage which can be caused to plants and to human and animal health.

For vegetation, some sensitive species show visible or physiological effects following exposure to 40 ppb or 50 ppb [3]. However, the effect of exposure can be modified by the presence of other atmospheric pollutants and, since ozone causes damage to vegetation through stomatal uptake, by nutritional status, light, temperature and humidity. There is genetic variability in the ozone response within species as well as between species and, although considerable attention has focused on crops and forests, little is known about the impact on semi-natural vegetation. Timing of the exposure within the life of the plant can be important as can be the time for recovery between exposures [6]. The United Nations Economic Commission for Europe is considering a tentative proposed critical level of 300 ppb • h above 40 ppb during daylight hours for the growing season of the vegetation. A critical level is defined as one below which ozone has no detectable effect. However, there are a number of outstanding issues which it is hoped to resolve by the end of 1993 and the adopted critical level may well be different. The proposed critical level would probably be exceeded in most of Europe at present.

Concern for human health in the UK at the current levels of ozone is growing but better assessments of population and individual exposure are thought necessary [6]. This aspect may in time be the main argument for emission controls of the major precursor gases.

It is important to differentiate between the dose which a plant or human receives, that is incorporated into the individual’s system by some method, and its exposure, that is the level in the atmosphere around the individual. In this paper current methods for determining plant exposure in the UK are described and then the potential application of extreme value theory is explored.

## 2. Ozone Exposure Maps of the UK

**Fig. 3:**
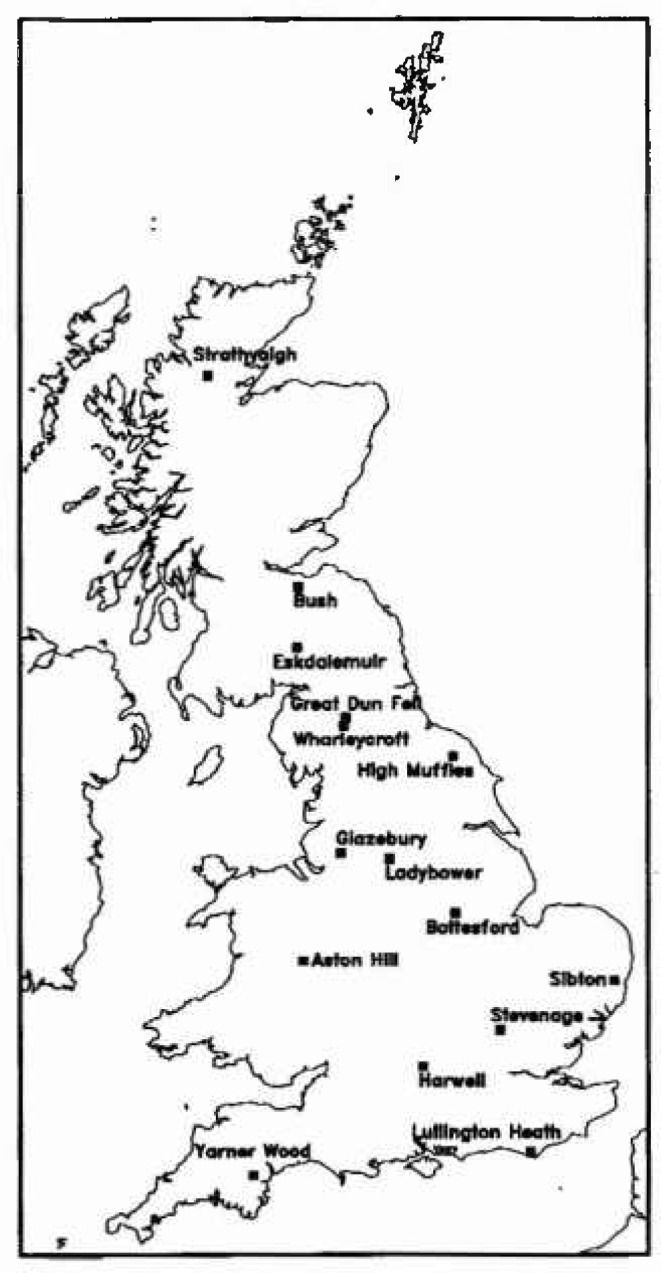
Locations of 15 monitoring sites on the UK mainland (2 sites, Lough Navar and Mace Head, are on Ireland).

**Fig. 4:**
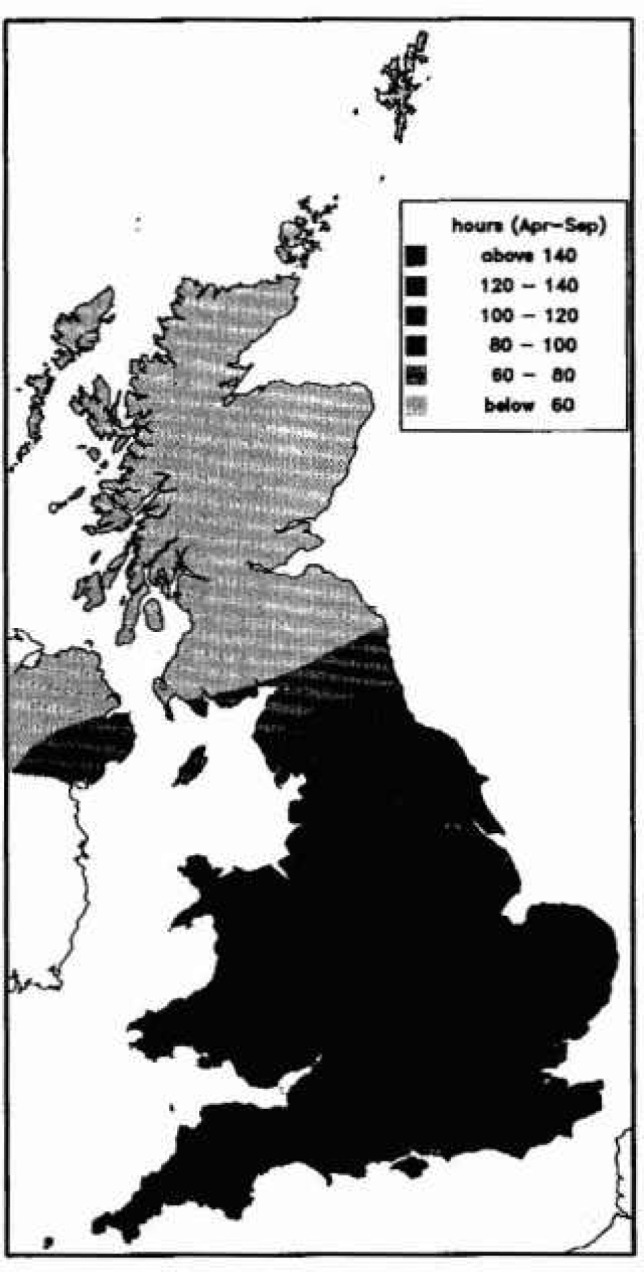
Interpolated map of the number of hours when ozone exceeds 60 ppb.

Ozone exposure has recently been mapped for the UK at three concentration thresholds: 40 ppb, 60 ppb and 90 ppb [5]. There were about 17 rural or semi-rural monitoring stations between 1987 and 1991 which recorded hourly mean concentrations in Britain and Ireland (Fig. 3). As the differences between sites which were geographically close was as large as the differences between geographically distant sites, a straight spatial interpolation between sites gave a map similar to that in Fig. 4.

**Fig. 5:**
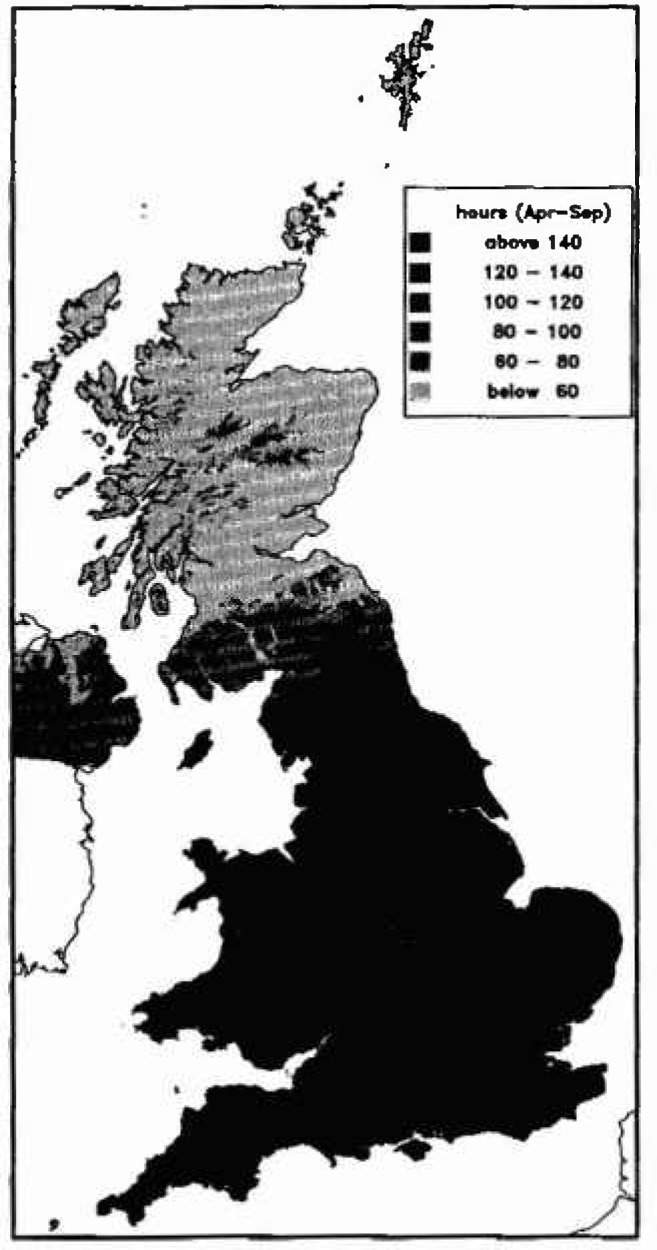
Altitude adjusted interpolated map of the number of hours when ozone exceeds 60 ppb.

In the summer months, taken as April to September, during the part of the day when the atmospheric boundary layer was well mixed by turbulence, ozone concentrations at neighbouring sites were very similar. For each threshold, an empirical relationship was derived between the hours over the threshold for the whole day and the hours over the threshold for the well mixed period, taken to be 1200 to 1800 GMT. For the threshold at 60 ppb, the relationship was

$$h_{60}=\left(1.3+0.0021\;z\right)t_{60,}$$ (1)

where *h*_60_ was the total hours over 60 ppb, *t*_60_ was the hours over 60 ppb between 1200 and 1800 GMT and *z* was the altitude of the location in meters. This relationship was applied to the spatial interpolation of hours over 60 ppb for 1200 to 1800 GMT to provide a map (Fig. 5) with clear topographical influence. The coastal effect, which can extend for 5 km to 20 km inland depending on meteorological conditions, was ignored; typically coastal ratios were around 2 rather than 1.3. The maps are only for the summer months, April to September, but ozone levels very rarely exceed 40 ppb during the remainder of the year.

This approach emphasizes the spatial variability of ozone exposure within small areas. The relationships for the different thresholds are empirical and must be recalculated for each threshold and they do not provide a general description of high concentration events. Direct estimates of exposure in terms of a dose measurement like ppb.hours are not available although a minimum estimate could be made. If the decision were made to set different windows to match the growing seasons of different vegetation types, the whole procedure could be difficult to implement.

## 3. Modelling Aggregate Excess

In work on flood levels for the River Thames, Anderson and Dancy [2] modelled the aggregate excess, that is the sum of the exceedances over a threshold, within a cluster using a Weibull distribution. There are similarities between ozone data and flood level data. A Pareto distribution has been shown to predict the peak excesses of ozone concentrations at a rural site using a threshold of 40 ppb [7]. There is a seasonal component in the data, since high concentrations rarely occur over the winter period, but this has not been modelled at present. Also ignored was the probable increase in mean values of ozone concentration over the time period of data collection, as this increase was small compared to both the diurnal fluctuations and the accuracy of the recording methods.

Anderson [1] has looked at ozone data for one site, Stevenage, for a longer time period. There was evidence of nonstationarity in that data set and he shows that there is a need for temperature, or some similar measure, as a covariate. This problem is still under investigation but for the time period considered in this paper, 1986 to 1991, no covariate has been used. Anderson also derived a method of extrapolating to higher thresholds than those used in fitting the models, a very useful tool for determining exposures to plants with different sensitivities to ozone.

The data for the 17 sites have been fitted using a single threshold of 40 nL/L and a Weibull distribution for the aggregate excess. The data were declustered using a minimum time separation of 48 hours. There were about 100 clusters for the sites with relatively complete data sets. Some sites were not operational in the earlier period of collection and one site had only 3 years data.

The two parameter Weibull model

$$p\left(S>s\right)=\exp\left(-\alpha \cdot s^{0}\right)$$ (2)

was fitted. The shape parameter, *θ*, varied between 0.4 and 0.6 for all sites. When *θ* was constrained to the value 0.5, there were only slight increases in the values of the likelihood function. The spatial variation was therefore explored using only the scale parameter *α*.

**Fig. 6:**
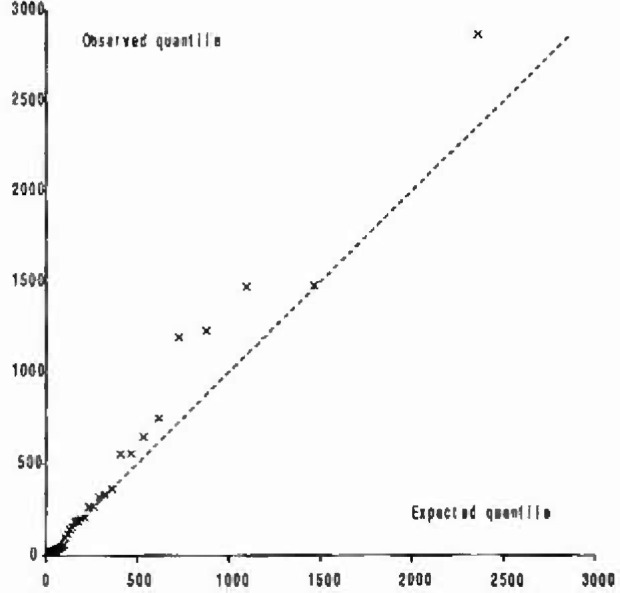
Q-Q plot for Weibull model (40 ppb threshold, 48 hour separation) fitted to data from Ladybower.

The Q-Q plots showed that, as expected, the fit of the Weibull model varied from site to site. There were some very straight line plots but there were also shapes typically illustrated by the plot for Lady-bower (Fig. 6). Most of the data were on a reasonably straight line but the Weibull distribution underpredicted a few data points, usually no more than five, at the higher values.

The shape parameter, *α*, from the fitted Weibull model was clearly related to a SE-NW trend across the country. To investigate this further, the *α* values were regressed on other available data.

The sites were referenced to a line from Lulling-ton Heath, a site on the south-east coast of England, to Strathvaigh in the north of Scotland using two variables, *n*_mod_, the distance north-west along the transect, and e_mod_, the perpendicular distance from the transect with positive values being to the east. The actual distances were divided by the length of the transect to give manageable numerical values. The relationship between *α* and *n*_mod_ was non-linear and the simplest function of *n*_mod_ which fitted well was *n*_mod_^4^. A linear function of *e*_mod_ improved the fit. One site, Bottesford, had a high residual. This site has peculiar local features which can give it the characteristics of an urban site and was removed from the data. The subsequent regression equation

$$\alpha =0.091+0.083\;{n_{\mathrm{mod}}}^{4}\;-0.023\;e_{\mathrm{mod}}$$ (3)

explained about 85% of the variation and produced an acceptable residual pattern. The 2 remote sites, Strathvaigh and Mace Head (on the west coast of Ireland) had the most influence on the fit.

When a threshold of 60 ppb was used, there were problems in fitting the model to the data. Each site had only about four independent clusters per year and fitting a Weibull model was very difficult since the likelihood surface was quite flat. Results were obtained by assuming *θ* was the same for a 60 ppb threshold as for a 40 ppb threshold. However, it became apparent that there were potentially two difference sources for the higher exceedances and that separation of these sources was critical with the increased threshold level.

## 4. Discussion

The results of these fits are encouraging although a number of problems have occurred. The Weibull distribution with a threshold of 40 ppb and *θ* Fixed at 0.5 gives an interpretable underlying pattern for the whole country. The SE-NW gradient would be expected. Areas to the east of the chosen transect are more influenced by air masses from continental Europe and would be expected to have more ozone episodes. The lack of detection of an altitude effect at this threshold is not entirely surprising as high altitude sites can have mean ozone concentrations quite close to this threshold. However an altitude effect would be expected at a higher threshold.

At the 60 ppb threshold two main problems occur. The first, and possibly the most important, is lack of data. The declustering algorithm which has been used takes the rather simple approach of removing clusters with missing data. When monitoring stations are running continuously, usually recording several times per minute, there are a whole series of glitches which can occur in the data for reasons wholly unconnected with the concentration values. In particular there may well be a series of instrumentation tests which usually occur during the working day and often at least once per week. Data capture rates of over 90 % on hourly values are regarded as good but not all sites on the network are achieving these rates. Therefore, careful decisions on the treatment of missing data are likely to give more information for analysis.

The second problem is one of determining whether there are two distinct distributions required to model threshold exceedances or whether the Weibull model is the wrong approach. If a meteorological covariate is introduced [1], it is not clear where it should be measured. Rapid ozone production can be occurring 50 km or 100 km downwind in good sunny conditions but the monitoring site may be sitting in quite a different climate. Clearly, further investigation of the peak and close to peak values will be required.

Although large exposure to ozone can be accumulated by a plant at concentrations over 100 ppb, these are relatively rare occurrences in the UK and are often, if not always, associated with very dry conditions. How much of the ozone will enter the plant’s system, given that the plant is probably under considerable water stress by the afternoon period, is not clear. Even if the models do not perform very well at the highest exceedances, if they can perform reasonably well for the remainder of the exceedances they could be of considerable benefit when critical levels for vegetation are considered. For human health problems, of course, a different perspective is required.

This approach, when combined with a model of time between clusters, has the potential of producing valuable information for the assessment and mapping of critical levels for vegetation. However, some further progress is required with models for the 60 ppb threshold and with identification of local scale variability in concentration levels.
